# Motivational Differences between Whole Blood and Apheresis Donors in Quebec, Canada: A Questionnaire-Based Survey in a Voluntary Nonremunerated Context

**DOI:** 10.1155/2015/568259

**Published:** 2015-07-29

**Authors:** Johanne Charbonneau, Marie-Soleil Cloutier, Élianne Carrier

**Affiliations:** Research Chair of Social Aspect of Blood Donation, INRS-Centre Urbanisation Culture Société, 385 Sherbrooke Street East, Montreal, QC, Canada H2X 1E3

## Abstract

*Background*. Finding ways to recruit apheresis donors is crucial. The aim of this study was to provide a quantitative analysis of the motivations of regular plasma/platelets donors (PPDs) in comparison with those of regular whole blood donors (WBDs), in a voluntary and nonremunerated context. *Study Design and Methods*. Motives to donate blood and demographic characteristics were collected through questionnaires completed by 795 WBDs and 473 PPDs. Chi-square tests were completed to determine which motivations stand out across the two blood donor groups. *Results*. The motivator selected by the highest percentage was “my blood can save lives.” Comparison of WBDs and PPDs showed that 12 out of 23 items were statistically significantly different from one group to another. *Conclusion*. The belief that helping others is in their nature is more prevalent among PPDs. In this sense, their profile is unique. Four other motivators distinguish this group from the WBDs: “I think there is a strong need for blood products,” “it gives me a sense of pride,” “I like to have goals,” and “I receive telephone reminders.” These motivators point to the role the ongoing support provided by blood collection agencies (BCAs) plays with PPDs.

## 1. Introduction

Finding effective ways to recruit blood donors is crucial for blood collection agencies (BCAs) given the rising demand for blood due to an aging population, strict donor deferral criteria, new therapeutic treatments, and the limited shelf life of blood products [1–3]. In addition, the demand for plasma-derived therapeutic products—particularly polyvalent intravenous immune globulin (IVIg)—is projected to increase in the future [4, 5]. However, apheresis components (plasma and platelets) are still procured from compensated donors in many high-income countries, such as Germany and the United States. This later country alone provides 55% of the world's supply of plasma derivatives [6]. As is the case in many countries, in the province of Quebec (Canada), the organization responsible for blood product supply, Héma-Québec, is attempting to increase its degree of self-sufficiency by appealing to nonremunerated volunteer donors [7]. Demand for IVIg in Quebec has increased by 8.3% annually since 2003 [8]. In 2013-2014, the rate of IVIg self-sufficiency was 14.5%. There are currently only five fixed collection sites equipped for apheresis donation in the province, two of which opened recently (in 2013). According to the most recent annual report, Héma-Québec will open new permanent blood centers devoted to the collection of plasma for fractionation in the next few years [8]. There are also plans for a fractionation plant to open in 2019 [9].

In such a context, recruitment to the plasmapheresis panel becomes a crucial issue. Apheresis donation differs in many ways from whole blood donation. Whereas a whole blood donor may donate once every 56 days, a plasma donor may donate every 6 days and a platelet donor every 14 days. In addition, plasma and platelet donation processes last longer due to the return of saline and red blood cells (RBC) to the donor: a minimum of 45 minutes for plasmapheresis and up to three hours for plateletpheresis as compared to up to 15 minutes for whole blood donation.

Despite the obvious need to recruit more apheresis donors, there is very little literature on their motivations. The meta-analysis review produced by Bednall and Bove in 2011 [10] on self-reported motivators for donating blood showed that only seven samples out of 92 included plasma or platelet donors. With only a single exception, all of the studies reported in their analysis were conducted in countries where remuneration for plasmapheresis or plateletpheresis is available.

Analyses of apheresis donors have focused primarily on their socioeconomic profile [11, 12], the conversion process from whole blood donation to apheresis donation [13–17], barriers (deterrents) or reasons given for discontinuing the practice [18–21], and, of course, the motivations of such donors, which have sometimes been compared to those of whole blood donors [11–13, 22–29].

Of the original eleven studies on donor motivators we identified, five had been conducted in the United States and the others in Australia or Europe (Germany, Austria, Belgium, France, or The Netherlands). In the three studies in which donors were remunerated [11, 26, 28], payment was the chief motivator for donation. Of the eight remaining studies, only three compared the motivations of apheresis donors with those of whole blood donors [12, 22, 27]. Retained samples for the eight studies ranged in size from 9 [23] to 2028 participants [22]. Three of the studies focused on platelet donors and the others on plasma donors. As might be expected, in smaller surveys, researchers chose to study motivations via semistructured interviews. In other studies, focus groups or mixed methods approaches with open- and closed-ended questions were used. Some researchers focused on the reasons behind the first apheresis donation, while others also explored general motivations (or the benefits donors perceived [13]). The number of answer choices given to participants in the four questionnaire surveys [22, 26–28] was quite limited (4, 6, 9, and 12). Finally, the analyses often focused on the primary motivator. However, qualitative studies on smaller sample using open-ended questions have shown the practice of blood donation to be associated with numerous motivators [23, 25], including personality traits, social influences, ongoing support by blood center staff, and the sort of practical accommodations agencies are able to provide donors with [30]. Moreover, the relative importance of different motivators changes over a donor's career [31]. Aside from the transition from extrinsic to intrinsic motivations [32, 33], the motivational dynamic is also linked to different events over the course of the donor's life [30]. For example, practicalities become a priority when donors must balance their donation practice with more demanding family and professional commitments.

In the studies selected by Bednall and Bove [10] as well as in those we found, the motivators most frequently cited by plasma and platelet donors were prosocial, even though such motivators would appear to reflect concerns that are more collectivistic (duty, obligation, and solidarity) than individualistic (altruism) in nature. Some studies highlighted the importance of the interpersonal relationships developed with collection staff and other donors [12, 13, 23, 25]. Other motivators associated with the specific conditions of apheresis donation were noted: the ease of developing a routine (due to the possibility of making an appointment) and the ability to increase one's number of donations or develop a sense of belonging to a special group, a blood donation “elite,” more rapidly. However, of the four studies that explored the range of apheresis donors' motivations in greater depth [13, 23–25], two made no comparisons with whole blood donors' motivations.

In short, studies discussed so far have numerous limitations. As noted by Bednall and Bove [10], few researchers have attempted to study apheresis donation in the context of volunteer nonremunerated donation. Samples have often been small, and few studies have compared the motivations of apheresis donors with those of regular whole blood donors. Analysis of motivations has itself often been limited in scope, due either to the restricted number of answer choices provided or to the researcher's decision to focus on the primary motivator. Bednall and Bove [10] also pointed out that most studies have focused on prosocial motivations and personal norms and shown little interest in social influences.

In response to these gaps in the literature, this study seeks to provide a thorough quantitative analysis of the motivations of apheresis donors (plasma/platelets) in comparison with regular whole blood donors, in the context of a voluntary and nonremunerated system.

## 2. Materials and Methods

### 2.1. Sample Selection

Sampling was done by accessing and extracting information from Héma-Québec's donor information system (Progesa, Mak System, Paris, France). Since 1987, this computerized database includes personal information on date of birth, sex, address of residence, dates of all previous donations, types of donations, previous and current deferrals, and screening test results for all donors. Two (a third group of lapsed donors were also recruited for this survey but were not used in the present analysis.) groups of donors were defined on the basis of their donation history: current whole blood donors (WBD) were those who had given two allogenic donations during their previous history and one donation during the 6 months preceding the survey, and plasma and platelets donors (PPDs) were those who had given three plasma or platelets donations during their previous history and one during the six months preceding the survey. Two other criteria were taken into account to extract a random sample of donors to whom the survey was sent: age (to be between 18 and 55 years at the time of the sample extraction) and sex (women were oversampled for PPD, since they are less often donors).

The initial targeted number of respondents was 750 subjects (50% men and 50% women): 500 regular WBDs and 250 regular PPDs. To achieve this target, 2000 WBDs were randomly selected from the database but the potential group of PPDs was smaller: the Progesa database contained 1968 donors who met our criteria (453 women and 1515 men). From this pool, all the women were selected and 547 men were randomly selected to achieve our target of 1000 mailed questionnaires. Participants' residential postal codes were used as geographical markers to ensure the sample's geographical representativeness. Sampling was conducted on March 25, 2014.

### 2.2. Questionnaire Development

Data were collected with a self-administered mailed questionnaire (see appendix). The survey was developed specifically for the study, which had a twofold topic: (1) motivators for blood donation and (2) the practical aspects of the blood donation experience. (When, where, and how long donors give blood; what forms of transportation they use; what activities precede or follow their visits to blood drives; what was the overall trajectory of their last visit; has their practice changed over the past few years and why; and diverse time pressure considerations.)

The first question concerned motivators for blood donation. Several sources were used in selecting and formulating the proposed answer choices (see Table 1). To begin with, our research team had completed four qualitative surveys of blood donors between 2009 and 2012 [30] (the methods used in the four surveys are presented in further detail in Charbonneau et al. [30]), conducting 136 interviews with WBDs and/or PPDs. We were inspired by the interdisciplinary perspective chosen by Piliavin and Callero [31] to study the career of blood donors. Our approach, incorporating sociological and psychological theories [34], sought to identify individual and social factors associated with the experience of giving blood. Although they had different objectives, all four surveys allowed for exploration of the reasons that had led donors to start their practice and to continue it, or not, over time. Respondents who remembered their first blood donation initially mentioned contextual elements that had contributed to it; those with long donor careers spoke of the circumstances that had enabled them to continue their practice. However, 85% of donors cited general motivations for their actions and always mentioned more than one. Although a respondent might say that a specific motivator was “the most important” (to meet the need for blood products, e.g.), later in the interview the same respondent would mention another “primary” motivator (e.g., the collection centre was close-by), when it came to mind in the course of the discussion. This multiplicity of motivators has been observed in other surveys [23, 25]; as suggested by Caillé [35], it lends itself to a combination of selfish reasons (egotism), selfless reasons (altruism and goodwill), and social obligations (social pressure), while always incorporating a number of institutional aspects (direct solicitation or ease of access to collection centers). Thus, in the present survey, respondents were given the opportunity to choose five answers out of 22 when asked about motives for donating blood. Furthermore, they were permitted to add their own reasons (the analysis of open-ended responses will be published later). They were not, however, asked to indicate a chief motive or to rate their answers.

In order to select answer choices for the present survey, we also reviewed the survey tools used by other researchers to study blood donation motivators, as well as of the results presented in 24 separate studies (see Table 1). Some statements—“someone close to me has received one or more blood transfusions in the past,” “it is my civic duty/a way to help out the community,” “I think there is a strong need for blood products”—have figured in a large number of studies. Others have been used less frequently—“I like to have goals (20, 50, 100, 200 donations, etc.),” “it is an activity that encourages you to monitor and take care of your health”—but were added because they tend to be cited more frequently in surveys with apheresis donors; this was also the case for “I join people that I know (donors, staff) at the blood drive.”

The second part of the questionnaire was also written based on the results of our previous qualitative studies. We also consulted other studies on the same topics before making the final decision as to the choice of questions. Questions on demographic characteristics (age, sex, education level, employment status, marital status, number of children, country of origin, postal code, and blood type) were also included, but they will be the subject of further analysis and are therefore only briefly exposed here.

The first version of the questionnaires was submitted to a pretest, involving 16 donors in four focus groups. Each focus group was designed to target a specific population (2 WBD groups, 1 PPD group, and 1 lapsed blood donors' group) and conducted in a different setting (Downtown Montreal (2), Southern Montreal Suburb, and Quebec City).

Groups ranged in size from 3 to 6 participants (27 to 53 years old; 7 women, 9 men). During each focus group, participants were first asked to complete the questionnaire. Each participant's responses were then shared with the group and discussed at length, one question at a time. The average focus group took approximately 1.5 hours. Each participant was given 20 CAN$ at the end of the focus group as a token of appreciation.

After the last focus group, the research team revised the questionnaire based on the participants' criticisms, comments, and suggestions. For the question on motivations, out of the original 24 statements, 11 remained the same, seven were reformulated, two were partially truncated, four were grouped together into two, and one was eliminated, while two new answer choices were added.

The final version of the questionnaire consisted of 25 questions and took approximately 20 minutes to complete. Two separate questionnaires were produced: one for the WBDs and one for the PPDs, where subjects were asked if they would like to participate in a qualitative follow-up survey (data to be analysed later). The questionnaires were first developed in French and then translated in English.

### 2.3. Survey Procedures

An introductory letter signed by an executive officer at Héma-Québec was included in the mailing package, along with a letter explaining the study signed by the head researcher, the questionnaire, the consent form, and a prestamped return envelope. Questionnaires were anonymous (no individual identifiers retained) but return envelopes were supplied with a code, permitting the questionnaire to be tracked back to the original database. The research ethics committees of the local university and Héma-Québec approved the study. Questionnaires were mailed based on language preference information in the donor information system (French = 6562 and English = 436). The initial questionnaire mail-out began on April 25, 2014. There were two successive waves in total (1000 PPD questionnaires mailed in May; 2000 WBD questionnaires mailed in July-August). We received the last completed questionnaire on January 28, 2015.

To avoid the possibility of analysis results being skewed due to the 22 answer choices to the motivation question being consistently presented in the same order, three versions of the questionnaire with different answer choice orders were produced and mailed out, with each version going to one-third of the two groups (WBD and PPD).

Of the 3000 questionnaires dispatched, 1361 were returned fully or partially completed. Forty-three (43) questionnaires were also returned owing to incorrect addresses, or because recipients had moved or were deceased. Ninety-three (93) questionnaires were eliminated from our analysis because they were returned without the consent form or had too much missing data. The response rate was 40% for the WBD group and 48% for the PPD group. As has been found elsewhere [36, 37], response rates were higher among women, especially for WBDs (46% versus 34% for men). Older donors (50–56 years old) had the highest response rates (11% for WBDs and 15% for PPDs) while the 30- to 39-year-old group had the lowest response rates both for WBDs (8%) and PPDs (9%), although differences with other age groups were small. Analyses of motivations were performed using 1268 admissible questionnaires (795 WBDs and 473 PPDs).

### 2.4. Statements' Classification and Statistical Analysis

For the purpose of this analysis, we classified the motivations' statements in the categories identified by Bednall and Bove in their meta-analysis [10] (see Table 1). These authors developed a taxonomy based on established terminology from both psychology [38] and marketing [39] literature. Sixteen out of 22 items from our questionnaire were easily classified in this taxonomy because our statement was referring to the same definition or example shown in Bednall and Bove's list. Three items fell under more than one category. For example, “helping other people is in my nature” could be classified as both a prosocial (altruistic) motivator and an intrinsic motivator. Likewise, “I like to have goals”—an item that, although not specifically mentioned in the list of Bednall and Bove [10], is more frequently cited by apheresis donors—may be interpreted as an intrinsic motivator that may be highly influenced by the recognition mechanisms established by BCAs. “I feel recognition from people around me” may belong as much in the social norms category, if friends and family members, as in the incentives category, express such recognition if it comes from BCAs instead. Three other answer choices were difficult to classify under the categories proposed by Bednall and Bove [10]. Saying that donating blood “is an activity that encourages monitoring and taking care of your health” does not entirely coincide with the notion of perceived health benefits. Also, while giving blood “is a positive thing to do and requires little effort” may be considered an altruistic motivator, the idea of a low cost charitable act also reveals a slightly egotistical nature. In the first version of the questionnaire, two separate statements were proposed, but the focus groups' participants suggested grouping them together. Finally, giving blood because the donor knows that “I have a rare and sought-after blood type” also lends itself to multiple interpretations (pride, a sense of obligation, and a need for recognition). This particular answer choice was not included in the Bednall and Bove [10] list, which instead contained “to learn my blood type,” a reflection of the fact that their meta-analysis also included studies on motivators for first donations. For the purpose of this analysis, each item was classified into only one category (which is shown in Table 1).

Data entry on ACCESS began in July 2014, alternating with mail-out of the final wave of questionnaires. Descriptive statistics were carried out for each question and demographic variable. Next, Chi-Two tests were completed to determine which motivations stood out across the three groups. Analyses were performed with SAS version 9.4.

## 3. Results

The final sample had more women in the WBD (58% women) but proportions of women and men were equivalent in the PPD group, as we had oversampled women (Table 2). Age groups were similar for WBDs and PPDs, with 25% of respondents belonging to the 18- to 29-year-old category, respectively, 22% and 19% of WBD and PPD respondents belonging to the 30- to 39-year-old category, almost a quarter of respondents to the 40- to 49-year-old (23% for WBDs and 25% for PPDs) a third to the 50- to 56-year-old age groups (30% for WBDs and 32% for PPDs). In terms of education and marital status, both groups were well educated (between 36% and 40% of respondents had university degrees) and the majority of respondents were married (63% and 64%). WBD respondents came mostly from Montreal (39% against 22% for PPDs) and elsewhere in Quebec (43% against 19% for PPDs). In part due to the small number of collection sites in the province, most PPDs came from the Quebec City region (59%). Only the gender and the region of origin variables were statistically different between the two groups.

Table 3 presents the proportion of respondents in the whole sample and the two subsamples (WBDs and PPDs) that selected each of the motivators. The motivator indicated by the highest percentage was “my blood can save lives,” with 81% of all respondents having included this motivation in their list and more WBDs (83%) than PPDs (77%). Comparison of WBDs and PPDs according to their reported motivators shows that results for 12 motivators were statistically significantly different from one group to another.

A greater proportion of WBDs selected “it is a positive thing to do and requires little effort” (62%), “I give thinking that a member of my family or a close friend could need blood someday” (47%), and “a blood drive is being held near where I live or near my workplace/place of study” (34%) as significant motivators. Three other motivators were more often cited by WBDs compared to PPDs, despite a lower proportion overall: “it gives me energy the following days” (5%), “when I see posters and advertising” (5%), and “my coworkers also give blood” (2%).

PPDs reported several motivators in greater proportions to WBDs. The highest proportion of PPD respondents was for the “Helping other people is in my nature” (61%) motivator. Almost half of them stated that “I think there is a strong need for blood products” (47%) and “it gives me a sense of pride” (45%) were important motivators. Finally, PPDs choose “I receive telephone reminders from Héma-Québec” (28%) and “I like to have goals” (23%) in greater proportions than WBDs.

Table 4 presents the proportion of respondents for each of Bednall and Bove [10] motivator categories. These categories combine all the motivators shown in Table 2. The category with the highest percentage is the “prosocial motivation” with 97% of both WBD and PPD respondents having selected at least one motivation in this category. The comparison of WBDs and PPDs according to their reported proportions of motivators in each category shows that results were statistically significantly different for five categories, four of them having higher proportions for PPDs. The only category where WBDs rated motivators in greater proportions is the “convenience of collection site” (34% against only 8% for PPDs). Approximately two-thirds of PPD respondents chose motivators in the “personal values” (61%) and “indirect reciprocity” (67%) categories, the highest proportions for both categories as compared to the WBDs (resp., 50% and 56%). Two other categories were higher for PPDs: the “perceived need for donation” (47%) and the “marketing communication” motivators (30%).

## 4. Discussion

Despite the growing demand for plasma-derived therapeutic products and several countries' desire to reduce their dependency on imported products, little research has been conducted to date on the behaviour of apheresis donors. This study sought to provide an analysis of the motivations of apheresis donors (plasma/platelets) in comparison with regular whole blood donors, in the context of a voluntary and nonremunerated system.

The results confirm those of other researchers who have highlighted the importance of prosocial motivations for blood donors. The greatest number of respondents, regardless of donor type, chose the altruistic motivation of “my blood can save lives.” Previous studies [22–25, 41, 42] have mentioned the importance of this motivation in blood donors. Although this formulation is not the one most commonly used in questionnaires for measuring prosocial altruistic motivation, it tends to be employed spontaneously by donors when answering open questions and in interview surveys [22, 23, 25, 30]. It also highlights the role of BCAs, which have long used it as a catchy promotional slogan. Whether given simply by reflex or because it is considered a socially desirable answer [10, 31], this response, which is strongly inspired and encouraged by the BCAs themselves, clearly promotes the utilitarian aspect of blood which does in fact save lives, in a very concrete fashion [30, 49].

If one applies the categories proposed by Bednall and Bove [10], one notes that almost all respondents (97%) mentioned one “prosocial motivation” or another. Motivations associated with “indirect reciprocity” were selected by the second highest percentage of respondents (60%). Donors were motivated to choose this category primarily due to someone close to them having received a transfusion. “Personal values” (“helping others is in my nature”) was the last category selected by more than 50% of respondents.

### 4.1. Different Motivations by Donor Type

The five answers selected by the highest percentages of respondents show that WBDs and PPDs are associated with different motivator groupings. Using the categories proposed by Bednall and Bove [10], we observe that for the two donor types the reasons for donating blood combine prosocial motivations, personal values, and the perception of need. But PPDs exhibit a greater range of motivators, encompassing indirect reciprocity in their grouping. Differences between the two profiles are also readily apparent when we analyze several of the possible motivators.

The second most popular motivator among WBDs is the belief that giving blood “is a positive thing to do and requires little effort.” Considering blood donation to be a positive gesture is also a prosocial altruistic motivation. However, the second part of the sentence lends itself to multiple interpretations. Given the fact that a very small proportion of the population gives blood, stating that donating blood is an act that requires very little effort may be a way of contradicting all those who cite numerous reasons for never giving blood. Further, from the perspective of an interpretation inspired by the sociology of gift-giving [50, 51], denying the effort demanded by this gesture is an expected reflex in the giving cycle, as it neutralizes the donor's sense of superiority towards the donation's recipient. However, emphasizing how little effort is required may also be interpreted as a reference to other types of charitable activities that call for a much more intensive form of commitment. From this perspective, blood donation suddenly seems to be a means of giving oneself a clear conscience “at little cost” to oneself [30]. Giving blood—which could take no more than fifteen minutes—once a year because a drive is held at one's workplace or place of study may be considered a fairly simple form of community service. However, our analysis reveals that a large proportion of apheresis donors also chose this statement, even though their practice is far more demanding in terms of frequency, distance traveled, and length of procedure [13].

Among PPDs, the second most popular motivator is the belief that “helping others is in my nature.” In their comparison of WBDs and plasma donors, Veldhuizen and van Dongen [27] suggested that “different donor profiles for whole blood and plasma donors already exist before the very first donation experience” (p. 1684). They observed that cognitive attitude was a better predictor of intention in future plasma donors than in WBDs and that “even before the first donation experience, future plasma donors were more motivated, felt more able to donate, and also expressed more positive attitudes toward donating blood” (p. 1683). In short, our results are in line with previous observations that suggest that PPDs are more “driven by self” [13, 24].

Four other motivators distinguish PPDs from WBDs: “I think there is a strong need for blood products,” “It gives me a sense of pride,” “I like to have goals,” and “I receive telephone reminders from Héma-Québec.” These motivators point to the role BCAs' ongoing support plays with PPDs. Staff members are the ones who let donors know that there is a strong need for plasma, that the act of donating plasma or platelets is rare, and also that these donors may feel being part of an elite [13, 25]. Blood collections have all, in their own way, developed recognition programs for major donors, which permit PPDs to enjoy high visibility, especially when they reach specific donation targets (100, 200, 500, etc.) Special gifts may mark the most memorable moments [52]. BCAs have also developed services that phone donors to remind them when the prescribed period between two donations is almost up, and to appeal directly to certain donors for targeted types of donations; many PPDs count on these reminders. These motivators call to mind Healy's assertion that “blood can be seen not as something that individuals donate but as something that organizations collect” [53, p.71]. These observations would tend to encourage the adoption of a sociological institutionalist approach [54–57], with its proposal that the institutional context gives rise to the conditions surrounding the practice of blood donation, as well as the rhetoric of altruistic donation, and that organizations contribute to defining a set of available and widely accepted reasons for donation, among which donors are able to pick and choose.

However, one could make the equally strong argument that BCAs' strategies are effective with a specific population that is particularly receptive to their messages, as is reflected in the previously cited results of Veldhuizen and van Dongen [27]. The results of their study suggest that plasma donors appear to be more conscientious than WBDs, having a personality-type generally characterized by the aim for achievement. The particular importance of indirect reciprocity for PPDs (“it gives me a sense of pride,” “I like to have goals”) also underscores the unique nature of a blood donation practice that is far more demanding than whole blood donation. When the personal cost is high, beliefs in personal benefits are more important for promoting actions [15, 44]. In short, donors who engage in this type of donation practice seem driven more by benevolence than by altruism [58].

What may be most surprising about our results is the low score the statement “I join people that I know (donors, staff) at the blood drive” obtained with the PPD group, as previous studies have emphasized the fact that such donors especially appreciate the possibility of developing personal bonds with collection staff and other donors who are present at the same time as them [13, 23, 25]. The low score may be due to the formulation of the statement chosen to express this phenomenon.

### 4.2. Recommendations

Some suggestions for recruiting and retaining plasma/platelet donors may be developed from these results. As we have observed, BCAs play a key role in supporting PPDs practices. In recent decades, there have been numerous studies in the field of behavioural psychology to determine the psychosocial factors that prompt individuals to develop a blood donation practice and maintain it over the long term [34]. Much less attention has been given to the influence of broader factors influencing blood donation, including the BCAs role [54]. First of all, collection staff is certainly in the best position to identify WBDs who display those characteristics most likely to promote conversion to apheresis donation, especially those individuals who seem interested in rapidly increasing their number of blood donations and who show considerable pride in reaching specific targets. Except for Schneider et al.'s [24], no study has so far pointed out the importance of achieving goals for PPDs.

Collection staff should also be strongly encouraged to spread information on apheresis donation, for example, by reminding people of the fact that the need is likely to grow while the country is far from self-sufficient as it is. As Kalargirou et al. [49] pointed out, this is not usually the strategy chosen by BCAs, which prefer focusing on altruism. Reminding PPDs that the organization is proud of them and that there are fewer apheresis donors than WBDs, making them truly special people, should also encourage donors. It is thus very important that recognition programs be maintained for this type of donor.

For its part, whole blood donation certainly benefits from the fact that it can be offered at mobile drives located as near as possible to donors' daily activities, which makes it a positive gesture that requires little effort. With attention given to the planning and development of new fixed centers for plasma, BCAs may be tempted to reduce the number of mobile drive locations. Some donors could then discontinue their practice if they are no longer able to find a place where they can give blood in their immediate environment.

### 4.3. Limitations

Our data have some limitations that must be discussed. First, our study was conducted on a sample of blood donors in Quebec, Canada, who may differ from blood donors in other countries. Moreover, we chose to restrict our study to donors between the ages of 18 and 55 years. The survey also tries to better understand the integration of blood donation into daily life and especially any barriers to the practice and reasons for its discontinuation among women in their thirties and forties: this is why our sample is age-specific. In addition, a complementary study using semistructured interviews was conducted to better understand the motivations and blood donation practice of PPDs; this second study involved an older population (up to 70 years of age) and results are to be published later. One should keep this possible bias in mind when discussing our data.

We should also point out the fact that we decided to place plasma and platelet donors together in the same group. While the growing need for blood products primarily concerns plasma derivatives, we believe that plasma and platelet donation practices and motivations are sufficiently similar for these two types of donors to be grouped together. Indeed, we know that donors increasingly make both plasma and platelet donations. It should also be noted that this grouping helped increase the number of potential respondents in our survey.

Our results were also constrained by the fact that motivations were self-reported, which may have biased results in favour of socially desirable answers [10, 13, 31]. Another of our study's limitation is that we did not specifically explore the motivations for first donations as other studies have done. This was because our previous studies [30] had demonstrated the difficulty of collecting reliable information on this topic when the first donation was several years prior, but it may account for why motivations associated with donors' social context—in particular, the influence of family—are less prominent in our results. We should also mention the nonstandard formulation of the statements chosen to represent the different motivators, which makes comparison of our results with those of other studies in the same domain somewhat difficult. Finally, we must remind the difficulty we had to classify some statements in the taxonomy of Bednall and Bove. Our results are directly influenced by the choices of classification we made.

## 5. Conclusion

At a time when numerous countries are seeking to develop increased self-sufficiency in plasma-derived products, it is extremely important to acquire a better understanding of the motivations of apheresis blood donors, especially in a context of voluntary nonremunerated context. Previous studies presented a detailed analysis of motivations for a small number of donors, or an analysis of motivations for a large number of donors, but only offering a very limited answer choice list. Our analysis is the first to combine a wide selection of blood donation motivators with a quantitative approach, therefore enabling the comparison of a large number of WBDs (795) and PPDs (473). Moreover, our survey permitted us to produce one of the very rare comparisons of WBDs, and PPDs in the domain. The decision not to have donors prioritize a single motivation permitted us to obtain a better understanding of the blend of motivators [30] that cause individual donors to give blood. These results offer points for BCAs to ponder in developing new blood donor recruiting and retention strategies.

## Supplementary Material

This questionnaire has been specifically developed by J. Charbonneau and M.S. Cloutier, for a study on “time use and blood donation” in Quebec, Canada. This study had a twofold topic: (1) motivators for blood donation and (2) the practical aspects of the blood donation experience.

## Figures and Tables

**Table 1:** Question on motivators for donating blood.

Time use and blood donation questionnaire Question 1 “which of the following elements motivate you to donate blood? (check off up to five answers)”	References	Motivator categories (Bednall and Bove [10])
A blood drive is being held near where I live or near my workplace/place of study	[18, 40]	Convenience of collection site

		Prosocial motivation
My blood can save lives	[18, 41, 42]	Altruism
It is a positive thing to do and requires little effort	[12, 18, 25, 40, 43]	
It is my civic duty/a way to help out the community	[18, 24, 25, 31, 40, 42, 44, 45]	Collectivism (community)
I give thinking that a member of my family or a close friend could need blood someday	[18, 23, 27, 40, 46]	Collectivism (family and friends)

		Personal values
Helping other people is in my nature^*∗*^	[18, 25, 44, 47]	Personal moral norms
My religious practice or convictions have encouraged me to donate	[18, 25]	Religiosity

I think there is a strong need for blood products	[12, 13, 18, 25, 40, 42, 45]	Perceived need for donation

		Indirect reciprocity
Someone close to me has received one or more blood transfusions in the past	[13, 18, 24, 25, 27, 40, 45]	Upstream
It gives me confidence that others will give if I need it later	[18, 23, 24, 40, 46]	Downstream
I like to have goals (20, 50, 100, 200 donations, etc.)^*∗∗*^	[24]	Intrinsic motivation
It gives me a sense of pride	[18, 25, 27]	Self-esteem

		Marketing communication
I receive telephone reminders from Héma-Québec	[18, 25, 27]	Direct marketing
When I see posters and advertising	[18]	Advertising
I join people that I know (donors, staff) at the blood drive	[13, 19, 20, 23, 25]	Blood drives

		Incentives
It is an activity that encourages you to monitor and take care of your health	[13]	Perceived health benefits
It gives me energy in the following days	[12, 18, 42]	
I have a rare and sought-after blood type	[40, 44, 48]	Learn blood type
I feel recognition from people around me^*∗∗∗*^	[13, 18, 25]	Recognition

		Social norms
Blood donation is a tradition in my family	[13, 18, 23, 25, 46]	Descriptive norm
My coworkers also give blood	[46]	Subjective norm
I like to be accompanied	[23]	

Another reason (specify):		

^*∗*^Also: intrinsic motivation (indirect reciprocity).

^*∗∗*^Also: recognition (incentives).

^*∗∗∗*^Also: subjective norm (social norm).

**Table 2:** Proportions of WBD and PPD respondents for each sociodemographic variable.

	Total	%	WBDs	%	PPDs	%	Sign. (*p*)
	*n* = 1268		*n* = 795				
Women	696	55	458	58	238	50	*p* < 0,05
Men	572	45	337	42	235	50	

18–29 years	317	25	200	25	117	25	n.s.
30–39 years	265	21	176	22	89	19	
40–49 years	300	24	182	23	118	25	
50–56 years	386	30	237	30	149	31	

Elementary/High School	295	23	199	25	96	20	n.s.
CÉGEP	498	39	311	39	187	40	
University	473	37	283	36	190	40	

Single	363	29	218	28	145	31	n.s.
Married, common law	804	64	506	65	298	63	
Divorced, separated, widowed	81	7	51	7	30	6	

Respondent from the Quebec City region	419	33	141	18	278	59	*p* < 0,0001
Respondent from the Montreal region	416	33	311	39	105	22	
Respondent from another region	433	34	343	43	90	19	

**Table 3:** Proportions of WBD and PPD respondents for each selected motivators.

Motivators	All donors	WBDs	PPDs	*P*
	(*n* = 1268)	(*n* = 795)	(*n* = 473)	
My blood can save lives	81%	83%	77%	<0,05
It's a positive thing to do and requires little effort	58%	62%	52%	<0,001
Helping other people is in my nature	53%	49%	61%	<0,0001
I give thinking that a member of my family or a close friend could need blood someday	44%	47%	40%	<0,05
I think there is a strong need for blood products	41%	38%	47%	<0,01
It gives me a sense of pride	38%	33%	45%	<0,0001
It's my civic duty/a way to help out the community	31%	32%	28%	NS
A blood drive is being held near where I live or near my workplace/place of study	24%	34%	8%	<0,0001
I receive telephone reminders from Héma-Québec	22%	18%	28%	<0,0001
I like to have goals (20, 50, 100, 200 donations, etc.)	17%	13%	23%	<0,0001
I have a rare and sought-after blood type	16%	17%	14%	NS
Someone close to me has received one or more blood transfusions in the past	16%	16%	16%	NS
It gives me confidence that others will give if I need it later	11%	12%	11%	NS
It's an activity that encourages you to monitor and take care of your health	8%	8%	9%	NS
Other reason	7%	6%	8%	NS
I feel recognition from people around me	6%	6%	7%	NS
Blood donation is a tradition in my family	6%	6%	6%	NS
It gives me energy in the following days	4%	5%	3%	<0,05
When I see posters and advertising	4%	5%	1%	<0,0001
My coworkers also give blood	2%	2%	1%	<0,05
I join people that I know (donors, staff) at the blood drive	2%	1%	2%	NS
My religious practice or convictions have encouraged me to donate	1%	1%	1%	NS
I like to be accompanied	0%	1%	0%	NS

**Table 4:** Proportions of WBD and PPD respondents for each of Bednall and Bove's motivator categories [10].

Motivator categories^*∗*^	All donors	WBDs	PPDs	*p* from Chi-Two
	(*n* = 1268)	(*n* = 795)	(*n* = 473)	
Convenience of collection sites	24%	34%	8%	<0,0001
Prosocial motivation	97%	97%	97%	NS
Personal values	54%	50%	61%	<0,0001
Perceived need for donation	41%	38%	47%	<0,01
Indirect reciprocity	60%	56%	67%	<0,0001
Marketing communication	25%	22%	30%	<0,01
Incentives	31%	31%	29%	NS
Social norms	15%	15%	14%	NS

^*∗*^See Table 1 for the list of motivators in each category.
