# Supplementary material for: Motivational Differences between Whole Blood and Apheresis Donors in Quebec, Canada: A Questionnaire-Based Survey in a Voluntary Nonremunerated Context
Source: J Blood Transfus. 2015 Jul 29;2015:568259. doi: 10.1155/2015/568259 (PMC4532863; doi:10.1155/2015/568259)
Supplement: Supplementary file 1 — This questionnaire has been specifically developed by J. Charbonneau and M.S. Cloutier, for a study on “time use and blood donation” in Quebec, Canada. This study had a twofold topic: (1) motivators for blood donation and (2) the practical aspects of the blood donation experience. [file 568259.f1.pdf]

INRS
Université d'avant-garde

TIME USE AND BLOOD DONATION

DONORS QUESTIONNAIRE

Johanne Charbonneau and Marie-Soleil Cloutier

Research Chair on the Social Aspects of Blood Donation

Guidelines to complete the questionnaire

1. It is important to answer all questions
2. The number of answers to check off is indicated in parentheses
3. There are no right or wrong answers
4. Answers will be kept confidential

SECTION 1. GENERAL EXPERIENCE WITH BLOOD DONATION

1. Which of the following elements motivate you to donate blood? (check off up to five answers)

☐ a) Helping other people is in my nature

☐ b) I give thinking that a member of my family or a close friend could need blood someday

☐ c) I receive telephone reminders from Héma-Québec

☐ d) I have a rare and sought-after blood type

☐ e) Blood donation is a tradition in my family

☐ f) It's my civic duty / a way to help out the community

☐ g) My blood can save lives

☐ h) I like to be accompanied

☐ i) When I see posters and advertising

☐ j) It gives me confidence that others will give if I need it later

☐ k) A blood drive is being held near where I live or near my workplace / place of study

☐ l) My religious practice or convictions have encouraged me to donate

☐ m) I think there is a strong need for blood products

☐ n) It's an activity that encourages you to monitor and take care of your health

☐ o) It gives me a sense of pride

☐ p) Someone close to me has received one or more blood transfusions in the past

☐ q) It gives me energy in the following days

☐ t) My coworkers also give blood

☐ u) I join people that I know (donors, staff) at the blood drive

☐ u) I like to have goals (20, 50, 100, 200 donation, etc.)

☐ u) It's a positive thing to do and requires little effort

☐ v) I feel recognition from people around me

☐ w) Other reason (specify): ____

2. In your opinion, which situation(s) best describe your practice of blood donation? (check off <u>up to three answers</u>)

- ☐ a) I donate blood as soon as I am eligible
- ☐ b) After a refusal, I donate blood as soon as I am eligible
- ☐ c) I donate blood when Héma-Québec has called to remind me to donate
- ☐ d) I donate blood when I don't have too many work- or school-related time constraints
- ☐ e) I donate blood when I don't have too many family-related time constraints
- ☐ f) I give when my health allows me
- ☐ g) I only or mostly donate blood when a mobile blood drive is organized in my religious/cultural centre, or by an association in which I am involved
- ☐ h) I only or mostly donate blood when a mobile blood drive is organized in my neighborhood/municipality
- ☐ i) I only or mostly donate blood when a mobile blood drive is organized at my workplace / place of study
- ☐ j) I donate blood when I feel like it, not as a result of specific constraints
- ☐ k) There are other circumstances that explain my blood donation frequency (specify):_________________________________

3. When in the week do you usually make your blood donation? (check off <u>one answer only</u>)

- ☐ a) Generally on weekdays (Monday to Friday)
- ☐ b) Generally on weekends
- ☐ c) Any time

4. When in the day do you usually make your blood donation? (check off <u>one answer only</u>)

- ☐ a) Relatively early in the morning
- ☐ b) Relatively late in the morning
- ☐ c) At lunchtime
- ☐ d) Relatively early in the afternoon
- ☐ e) Relatively late in the afternoon
- ☐ f) In the evening
- ☐ g) No time in particular

5. On average, how long does your blood donation take (from your arrival to the time you leave)?

- ☐ a) Less than 30 minutes
- ☐ b) From 30 to 44 minutes
- ☐ c) From 45 to 60 minutes (one hour)
- ☐ c) From 61 to 89 minutes
- ☐ d) More than 90 minutes (one hour and a half)

6. **What means of transportation do you usually use to go donate blood? (check off <u>one answer only</u>)**

☐ a) None (takes place at work/school)

☐ b) Active transportation (on foot, by bike)

☐ c) Public transportation (bus, subway)

☐ d) Car

7. **Which of the following best describes the location of the blood drive that you attend <u>most often</u>?**

☐ a) The blood drive is near my home

☐ b) At my work/school or near my work/school location

☐ c) The blood drive is on the way between my home and my work

☐ d) The blood drive is near activities that I regularly attend

☐ e) I make a fairly long trip specifically in order to go to the blood drive

☐ f) None of the above

8. **Usually, when you go to donate blood, do you take advantage of the opportunity by combining donation with other activities? (check off <u>one answer only</u>)**

☐ a) yes

☐ b) no (Go to question 10)

9. **If you answered yes to the previous question, which type(s) of activity do you usually combine with your practice of blood donation? (check off all answers that you consider relevant)**

☐ a) Shopping

☐ b) A social activity with family or friends

☐ c) A volunteer or religious activity

☐ d) Activities on the way to or from the blood drive, before or after donating blood

☐ e) Other (specify): ____

10. **The experience of your latest donation**

10.1. If we take the example of your latest blood donation, which means of transportation did you use to go donate blood? (check off <u>one answer only</u>)

☐ a) None (at work/school) (Go to question 11)

☐ b) Active transportation (on foot, by bike)

☐ c) Public transportation (bus, subway)

☐ d) Car

10.2. If you checked off b), c) or d) in the previous question, from where did you leave in order to go make your blood donation? (check off <u>one answer only</u>)

☐ a) Home

☐ b) Work/school

☐ c) Leisure activity

☐ d) Other (specify): ____

10.3. If you checked off b), c) or d) in question 10.1, in your opinion, how far was the blood drive from your previous location? (check off one answer only)

☐ a) Less than 5 km

☐ b) Between 6 and 15 km

☐ c) Between 16 and 30 km

☐ d) More than 30 km

11. Changes in blood donation practices over the past five years

11.1. Within the last 5 years, have you made blood donations less frequently? (check off one answer only)

☐ a) yes

☐ b) no (Go to question 12)

11.2. If you answered yes to the previous question, would you say that this lower frequency of blood donation is related to some of the following reasons? (check off all answers that you consider relevant)

☐ a) Time constraints related to work or studies

☐ b) Time constraints related to family responsibilities or taking care of children

☐ c) Time constraints related to leisure or sport activities

☐ d) Time constraints related to volunteer activities

☐ e) Exclusion(s) as a result of traveling abroad

☐ f) Health reasons related to childbirth (pregnancy, breast-feeding)

☐ g) Other health reasons

☐ h) Moving or being farther away from the blood drive you used to attend

☐ i) Fear of needles or blood

☐ j) A painful or difficult experience with having your blood drawn

☐ k) The absence of a blood drive near the places that you regularly frequent

☐ l) The difficulty of accessing the blood drive (parking or public transportation)

☐ m) Volunteer or staff behaviour at the blood drive

☐ o) Too much time spent waiting or donating blood

☐ p) Not enough information on blood drive locations and schedules

☐ q) Blood drive too restrictive schedule

☐ r) Loss of interest in this cause

☐ s) Forgetting to donate or not receiving a telephone reminder from Héma-Québec

☐ t) Other reason (specify): ____

☐ u) No reason in particular

Section 2. Relationship to time in everyday life

IN THE FOLLOWING SECTION, WE INVITE YOU TO THINK ABOUT YOUR SITUATION IN LIFE GENERALLY AND NOT ONLY IN RELATION TO YOUR BLOOD DONATION EXPERIENCE

12. Do you feel under time pressure? (check off one answer only)

☐ a) Yes, every day

☐ b) Yes, several times per week

☐ c) Yes, about once per week

☐ d) Yes, about once per month

☐ e) No (seldom or never)

13. Do you experience free time moments which allow you to make spontaneous activities? (check off one answer only)

☐ a) Yes, every day

☐ b) Yes, several times per week

☐ c) Yes, about once per week

☐ d) Yes, about once per month

☐ e) No (seldom or never)

14. On a scale from 1 to 10, 1 being "people who have nothing to do and have all the time in the world" and 10 being "people who are always busy and never have any spare time", how would you rate yourself? (circle your answer)

1	2	3	4	5	6	7	8	9	10

15. If you could change one of the following schedules, which one would you change? (check off up to three answers)

☐ a) my work schedule

☐ b) the schedules of the public transportation I use

☐ c) my spouse's work schedule

☐ d) the opening and closing hours of administrative services or store

☐ e) my children's school or day care schedule

☐ f) blood drive schedules

☐ g) other (specify):____

☐ h) none of the above; all of these schedules are perfectly fine for me as they are

Section 3. Individual characteristics

16. How old are you? _________ years old

17. What is your sex?

☐ a) Female ☐ b) Male ☐ c) Other

18. What is the highest education level you have completed (degree obtained)?

☐ a) Primary

☐ b) Secondary / High School

☐ c) College (or equivalent)

☐ d) University undergraduate

☐ e) University graduate

19. What is your current employment status? (check off all answers that apply)

☐ a) Full-time worker

☐ b) Part-time worker

☐ c) Full-time student (Go to question 20)

☐ d) Part-time student (Go to question 20)

☐ e) Experiencing a temporary work interruption (parental leave, sick leave or other) (Go to question 20)

☐ f) Unemployed or between jobs (Go to question 20)

☐ g) Retired or no longer in business (Go to question 20)

☐ h) Housewife or househusband (Go to question 20)

☐ i) Other (specify):_______________________ (Go to question 20)

19.1. If you answered a) or b) to the previous question, which of the following best describes your work hours? (check off one answer only)

☐ a) During the day, Monday to Friday

☐ b) In the evening, Monday to Friday

☐ c) At night, Monday to Friday

☐ d) Alternating (day/evening/night)

☐ e) Combination of evening/weekend

☐ f) Other (specify): _________________________

20. What is your current marital status?

☐ a) Single

☐ b) Married or common-law spouse

☐ c) Divorced, separated or widowed

21. Do you have children?

☐ a) Yes

☐ b) No (Go to question 23)

22. If you answered a) to the previous question,

22.1. How many children live in your household? _______________ (if answer = 0, go to question 22.3)

22.2. How old are the children living in your household?

Child 1: _______ years old
Child 2: _______ years old
Child 3: _______ years old
Child 4: _______ years old
Child 5: _______ years old

22.3. Do you need to drop off one or more children at school or at daycare on the way from home to work?

☐ a) Yes, every day

☐ b) Yes, occasionally

☐ c) No (or not applicable)

23. What is your country of birth? _________________________

24. What is your blood type?

☐ A positive

☐ A negative

☐ B positive

☐ B negative

☐ AB positive

☐ AB negative

☐ O positive

☐ O negative

☐ Don't know

25. What is your postal code? ___ ___ ___ ___ ___ ___
